# Using fMRI to examine nonlinear mixed selectivity tuning to task and category in the human brain

**DOI:** 10.1162/imag_a_00354

**Published:** 2024-11-08

**Authors:** JohnMark Taylor, Yaoda Xu

**Affiliations:** Zuckerman Mind Brain Behavior Institute, Columbia University, New York, NY, United States; Department of Psychology, Yale University, New Haven, CT, United States

**Keywords:** dorsal, ventral, fMRI, task, mixed selectivity, vision

## Abstract

Recent experimental and theoretical work has shown that nonlinear mixed selectivity, where neurons exhibit interaction effects in their tuning to multiple variables (e.g., stimulus and task), plays a key role in enabling the primate brain to form representations that can adapt to changing task contexts. Thus far, all such studies have relied on invasive neural recording techniques. In this study, we demonstrate the feasibility of measuring nonlinear mixed selectivity tuning in the human brain noninvasively using fMRI pattern decoding. To do so, we examined the joint representation of object category and task information across human early, ventral stream, and dorsal stream areas while participants performed either an oddball detection task or a one-back repetition detection task on the same stimuli. These tasks were chosen to equate spatial, object-based, and feature-based attention, in order to test whether task modulations of visual representations still occur when the inputs to visual processing are kept constant between the two tasks, with only the subsequent cognitive operations varying. We found moderate but significant evidence for nonlinear mixed selectivity tuning to object category and task in fMRI response patterns in both human ventral and dorsal areas, suggesting that neurons exhibiting nonlinear mixed selectivity for category and task not only exist in these regions, but also cluster at a scale visible to fMRI. Importantly, while such coding in ventral areas corresponds to a rotation or shift in the object representational geometry without changing the representational content (i.e., with the relative similarity among the categories preserved), nonlinear mixed selectivity coding in dorsal areas corresponds to a reshaping of representational geometry, indicative of a change in representational content.

## 1 Introduction

In everyday vision, information is always encoded in a particular task context, whether we are simply browsing or looking for something specific. Recent experimental and theoretical work has shown that nonlinear mixed selectivity, where neurons exhibit interaction effects in their tuning to multiple variables (e.g., stimulus and task), may help enable the primate brain to form representations that can adapt to changing task contexts (Bernardi et al., 2020;Fusi et al., 2016;Rigotti et al., 2013). Compared with neural codes involving either pure selectivity or linear mixed selectivity (i.e., neurons tuned to just one variable, or neurons tuned to multiple variables in a purely linear manner), nonlinear mixed selectivity tuning can support a wider array of readout functions (Bernardi et al., 2020;Fusi et al., 2016;Rigotti et al., 2013).

Broadly speaking, nonlinear mixed selectivity simply refers to an interaction effect between two variables in a measured neural response (i.e., where a neural response to a combination of features cannot be modeled as the sum of its responses to the individual features, corresponding to a significant interaction effect in an ANOVA), but such interaction effects can take various forms. One familiar example is the concept of a “grandmother” cell that responds to one and only one combination of features (e.g., to the conjunction of “female,” “actress,” and “blonde-haired,” but not to any of these properties individually), such as “Jennifer Aniston” neurons in the hippocampus (Quiroga et al., 2005), neurons tuned to particular conjunctions of color and form (Seymour et al., 2010;Taylor & Xu, 2022), or neurons that only respond to a particular combination of stimulus and task, but the concept is broader than this. For example, neurons in the macaque prefrontal cortex (PFC) have been shown to exhibit heterogeneous yet stable response profiles to seemingly random nonlinear combinations of objects and task contexts (Rigotti et al., 2013). Such neuronal response profiles are believed to greatly increase our mental capacity to represent unique combinations of objects and task contexts as well as any combination of input variables. Nonlinear mixed selectivity tuning has been found in frontal cortex and hippocampus to represent combinations of stimulus and task variables (Bernardi et al., 2020,Dang et al., 2021;Grunfeld & Likhtik, 2018;Parthasarathy et al., 2017;Rigotti et al., 2013), in mouse entorhinal cortex to encode combinations of navigation-relevant variables (Hardcastle et al., 2017), and in posterior parietal cortex (PPC) to represent combinations of motor variables (Zhang et al., 2017,^2020^). However, thus far such studies have largely used invasive electrophysiological recording techniques in either nonhuman primates or opportunistically in special human populations (e.g., presurgical patients with implanted intracranial electrodes). If nonlinear mixed selectivity coding plays an important role in mental representation, it would be desirable to develop analysis approaches that can probe such coding noninvasively using techniques such as fMRI in the human brain. An added advantage of fMRI is that it allows multiple brain areas to be examined and compared within the same participants, compared with the limited spatial coverage of neurophysiological studies. In an earlier study, we developed a technique called*pattern difference decoding*to measure nonlinear mixed selectivity to color and form in the human brain, but thus far this technique has only been applied to stimulus variables, and not to more cognitive variables such as task context (Taylor & Xu, 2022). The central goal of the present study, therefore, is to examine whether it is indeed feasible to use fMRI to examine nonlinear mixed selectivity tuning to stimulus and task in the human brain. Since nonlinear mixed selectivity can affect representational geometry in different ways (e.g., by rotating a geometry for different contexts without changing the distances, versus actually changing the distances), a further goal of this study is to examine how nonlinear mixed selectivity modulates representational geometry.

Previous fMRI studies have shown that visual object representations in the human PPC are strongly modulated by task context, whereas those in occipitotemporal cortex (OTC) are more invariant to task (Bracci et al., 2017;Vaziri-Pashkam & Xu, 2017;Xu & Vaziri-Pashkam, 2019). Notably, these studies have largely compared tasks that vary the input of visual processing by manipulating spatial, object-based, or feature-based attention (e.g.,Vaziri-Pashkam & Xu, 2017). While attention is an important task factor, showing that a brain region responds differently to different visual input as determined by attentional selection may not be surprising. Thus, in line with other studies of mixed selectivity that employ pairs of tasks with similar attentional demands while varying subsequent cognitive operations (Bernardi et al., 2020;Rigotti et al., 2013), in the present study we use two tasks that attempt to equate the overall attended visual features while varying downstream information manipulation, allowing us to extend previous fMRI work and examine how task manipulations with matched attentional demands affect PPC visual representations.

## 2 Materials and Methods

In the present study, we asked participants to attend to object shapes while performing two different tasks that were similar in their overall attended visual features, but different in terms of the subsequent cognitive operations involved. In one task, participants responded if the exact same stimulus exemplar repeated twice in a row, while in another task they responded if the category of a stimulus did not match that of the surrounding block. We then performed a series of multivoxel decoding analyses to characterize how object category and task are jointly represented in early visual, ventral, and dorsal brain regions.

### 2.1 Participants

A total of 13 healthy adults (7 females) participated in the study; all had normal color vision and normal or corrected-to-normal visual acuity, and were between 18 and 35 years old. This sample size is approximately double the sample size (N = 7) used in other recent studies examining the task modulation of object representations in the human visual system (Vaziri-Pashkam & Xu, 2017,^2019^;Xu & Vaziri-Pashkam, 2019), and nearly triple the sample size (N = 5) used in two studies examining color–shape and color–motion interaction effects in visual cortex (Seymour et al., 2009,^2010^), in order to increase power. All participants gave their written informed consent before the experiments and received payment for their participation. The experiments were approved by the Committee on the Use of Human Subjects at Harvard University.

### 2.2 Stimuli

**Fig. 1.**
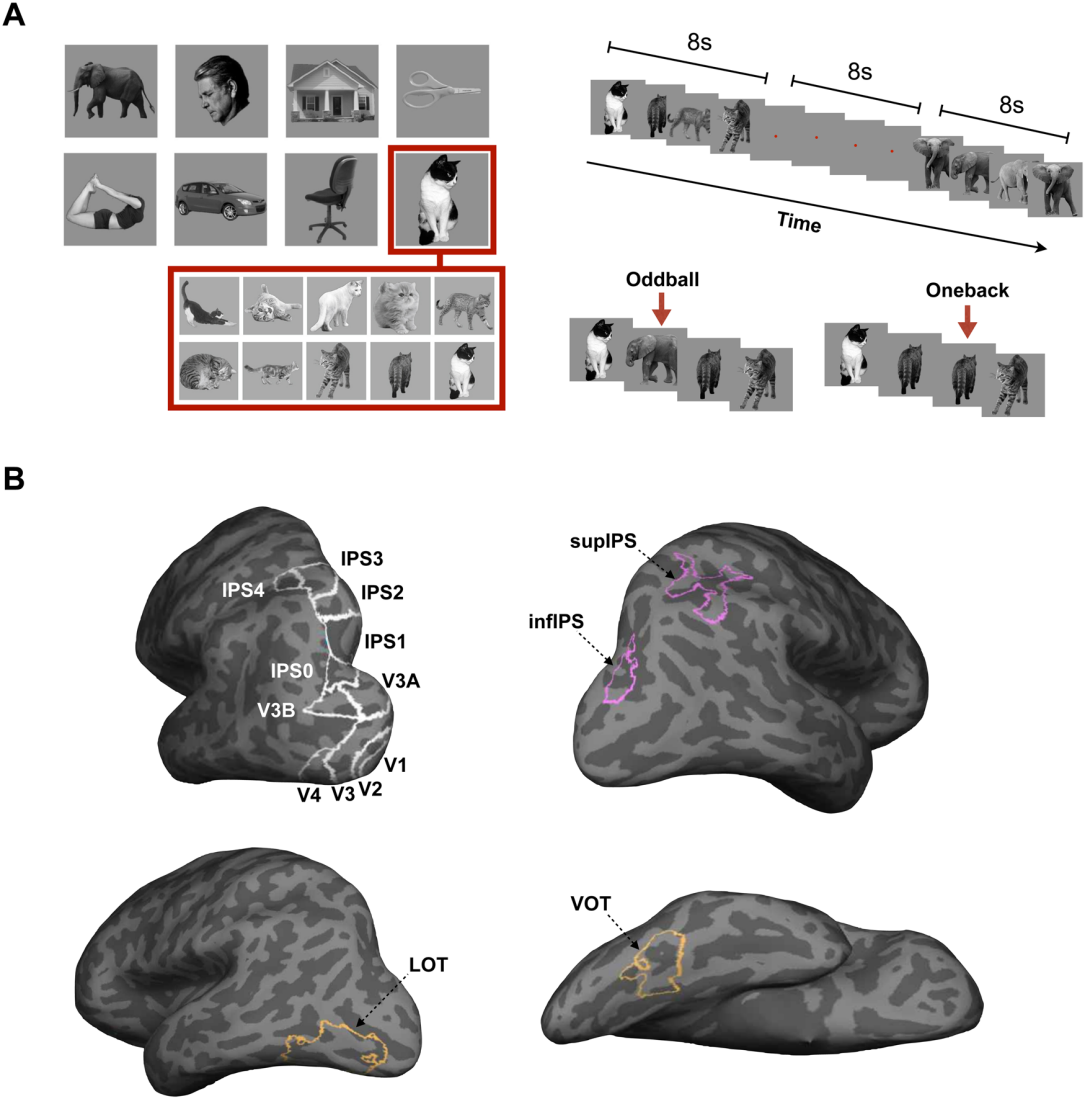
Stimuli, regions of interest, and methods. (A) Participants viewed blocks of stimuli drawn from eight different categories, where individual exemplars varied based on identity, pose, and expression within each category. Participants performed either an Oddball task, in which they responded with a button press if the category of a stimulus did not match the other stimuli in the same block (e.g., an elephant among cats), or a Oneback task, in which they responded if exactly the same stimulus exemplar was repeated twice in a row. (B) Regions of interest used in the experiment, with examples shown on the inflated cortical surfaces of example participants.

As stimuli, we used grayscale object images from eight different categories: faces, bodies, houses, cats, elephants, cars, chairs, and scissors (Fig. 1A). These stimuli were drawn from previous studies examining object and task representation in the human visual system (Vaziri-Pashkam & Xu, 2017,^2019^;Xu & Vaziri-Pashkam, 2019). Categories were chosen to cover a broad range of the natural visual categories encountered in our everyday environment and were typical of the categories used in previous investigations of object category representations in ventral visual cortex (Haxby et al., 2001;Kriegeskorte et al., 2008). For each object category, 10 unique stimulus exemplars were selected. These exemplars varied in identity, pose (for cats and elephants), expression (for faces), and viewing angle to reduce the likelihood that object category decoding would be driven by the decoding of any particular exemplar. Objects were placed on a light gray background and subtended 9.24° of visual angle. Participants were instructed to view the images while fixating at a centrally presented red dot subtending 0.46° of visual angle.

### 2.3 Procedure

Stimuli were presented in a block design (Fig. 1A). In each block, 10 exemplars from the same object category were presented sequentially, each for 200 ms followed by a 600 ms fixation period between the images. In the beginning of each run, participants were instructed to perform one of two different tasks. In the Oddball Task, participants responded if a given object exemplar did not match the category of the current block (e.g., if there was a cat in a block of elephants). The Oddball stimulus never occurred earlier than the third trial in the sequence of 10 trials in a given block, so as to allow the participant to establish the correct category of the block. In the Oneback task, participants responded if there was a back-to-back repetition of the exact same object exemplar (e.g., the exact same face twice in a row). Each run contained 10 blocks. Eight of these blocks did not contain the task (i.e., they contained neither an Oddball nor a Oneback trial), and two did; however, the participant did not know in advance which would contain the task, such that they had to remain prepared to respond during the whole run. The two blocks containing the task responses were discarded during data analysis to remove task response-related neural responses; thus, the blocks that were actually analyzed were visually identical between the two tasks. The eight blocks without the task spanned all eight stimulus categories. The two blocks with the task each contained a randomly chosen category, with the constraint that the category chosen differed from the categories used two blocks before and after that block.

Each of the 10 blocks in each run lasted 8 s, with a 12 s fixation before the first block and an 8 s fixation between blocks, for a total run duration of 2 min 52 s. Participants completed two practice blocks prior to the first TR being collected to remind them of the task for that run, while the scanner reached equilibrium. Each participant performed a total of 20 runs, with 10 runs in each task. The task alternated each run with the exception of the 10th and 11th runs, which had the same task, ensuring that no task was presented earlier or later on average than the other task, which might have introduced confounds due to scanner drift and/or participant fatigue. The two possible task orders were counterbalanced across participants. To determine the block order in each run, a 10 x 10 balanced Latin square was randomly generated for each task for a given participant, where numbers 1–8 (one for each category) corresponded to blocks without the task and 9–10 corresponded to task blocks. This balancing scheme ensured that all categories and task blocks occurred equally often in each presentation position of a run.

### 2.4 Localizer experiments

As regions of interest (Fig. 1B), we included retinotopically defined regions V1, V2, V3, and V4 in early visual cortex, the dorsal stream retinotopic maps V3A/B, IPS0, IPS1, IPS2, IPS3, and IPS4, two functionally defined IPS subregions that have previously been shown to be involved in object selection (inferior IPS) and storage (the superior IPS), and functionally defined regions in lateral and ventral occipitotemporal cortex (LOT and VOT) showing univariate sensitivity to object shape (Grill-Spector et al., 2001;Kourtzi & Kanwisher, 2001;Xu & Chun, 2006). All the localizer experiments conducted here used previously established protocols, and the details of these protocols are reproduced here for the reader’s convenience.

To localize topographic visual field maps, we followed standard retinotopic mapping techniques (Sereno et al., 2001). Specifically, a 72° polar angle wedge swept either clockwise or counterclockwise (alternating each run) across the entire screen, with a sweeping period of 36.4 s and 10 cycles per run. The entire display subtended 23.4 × 17.6° of visual angle. The wedge contained a colored checkerboard pattern that flashed at 4 Hz. Participants were asked to detect a dimming that could occur anywhere on the polar angle wedge. Each participant completed 4–6 runs, each lasting 376 s.

We localized two shape regions in lateral occipitotemporal (LOT) and ventral occipitotemporal (VOT) cortex, following the procedure described byKourtzi and Kanwisher (2001), and subsequently used in several of our own studies (Taylor & Xu, 2022;Vaziri-Pashkam & Xu, 2017,^2019^). LOT and VOT approximately correspond to the locations of LO and pFs (Kourtzi & Kanwisher, 2001;Malach et al., 1995;Vaziri-Pashkam & Xu, 2017;Xu & Vaziri-Pashkam, 2019) but extend further into the temporal cortex in an effort to capture the continuous activations often seen extending into the ventral temporal cortex. Additionally, we localized the inferior IPS, a dorsal stream region that past work suggests is involved in object selection (Xu & Chun, 2006). Specifically, in a separate scanning session from the main experiment (usually the same one as the retinotopic mapping session), participants viewed grayscale pictures of faces, places, common objects, arrays of four objects, phase-scrambled noise, and white noise in a block design paradigm, and responded with a button press whenever the stimulus underwent a slight spatial jitter, which occurred randomly twice per block. Each block contained 20 images from the same category, and each image was presented for 750 ms each, followed by a 50 ms blank display, totaling 16 s per block, with 4 blocks per stimulus category. Each run also contained a 12 s fixation block at the beginning, and an 8 s fixation block in the middle and end. Images subtended 9.5° of visual angle. Participants performed either two or three runs, each lasting 364 s.

To identify superior IPS, we used a visual short-term memory (VSTM) paradigm first developed byTodd and Marois (2004). Two slightly different versions of this localizer were used across participants: seven participants completed one version and the remaining six completed the other version (due to refinement of the localizer paradigm over time; no significant or trending difference was found between the ROIs localized using these two versions of the localizer in any of the analyses in this study). Both versions used an event-related design (as inJeong & Xu, 2016) where participants viewed a sample display of several objects and, after a delay, judged whether a new probe object matched one of the sample objects. In the first version, the sample display included 1–4 everyday objects, and the probe object appeared in the same location as one of the sample objects. A match occurred in half of the trials. Objects were gray-scaled images from four categories (shoes, bikes, guitars, and couches). In the sample display, objects could be placed above, below, to the left, or to the right of the central fixation ~4.0° away from the fixation (center to center). Four dark-gray rectangular placeholders, subtending 4.5° × 3.6°, marked all the possible object positions and were always present during the trial. The entire display subtended 12° × 12°. Each trial lasted 6 s and consisted of a fixation period of 1000 ms, a sample display period of 200 ms, a delay of 1000 ms, a test display period of 2500 ms in which participants provided their responses, and a feedback period of 1300 ms. Each run contained 15 trials for each set size and 15 fixation trials in which only the fixation dot appeared for 6 s. The trial order was predetermined using a counterbalanced trial history design (Todd & Marois, 2004;Xu & Chun, 2006). Two filler trials appeared at the beginning and one at the end of each run for practice and trial history balancing purposes. Each participant completed two runs of this localizer run, each lasting 8 min.

The alternative localizer was largely similar, with a few small changes. The set size varied from one to six instead of from one to four to allow more fine-grained measurement of performance at higher set sizes. Seven, rather than four, object categories were included, and they were couches, lamps, guitars, scissors, shoes, teapots, or umbrellas. Sample objects could be present in one of eight, rather than just four, locations (top, bottom, left, right, or one of the four intermediate diagonals, forming an octagon of possible placements). The rectangular gray placeholders were not used. Sample stimuli were now presented sequentially, rather than simultaneously, over the course of 1200 ms, each for a 100 ms slot randomly inserted into this 1200 ms window (50 ms between slots). The retention period lasted 950 ms, the test display lasted 2000 ms (instead of 2500 ms), the feedback period lasted 1600 ms (instead of 1300 ms), and the fixation period lasted 250 ms; thus, the total trial length was still 6 s. The probe object now appeared in the center of the display, rather than in one of the positions previously occupied by one of the objects. Participants completed three runs of this localizer, each lasting 8 min as in the other version.

### 2.5 MRI methods

MRI data were collected using a Siemens PRISMA 3T scanner, with a 32-channel receiver array head coil at the Harvard Center for Brain Science. Participants lay on their backs inside the MRI scanner and viewed the back-projected display through an angled mirror mounted inside the head coil. The display was projected using an LCD projector at a refresh rate of 60 Hz and a spatial resolution of 1024 × 768. An Apple Macbook Pro laptop was used to generate the stimuli and collect the motor responses. All stimuli were created using MATLAB and Psychtoolbox (Brainard, 1997), except for the topographic mapping stimuli which were created using VisionEgg (Straw, 2008).

A high-resolution T1-weighted structural image (1.0 × 1.0 × 1.3 mm) was obtained from each participant for surface reconstruction. For the first version of the superior IPS localizer scans, 24 axial slices parallel to the AC-PC line (5 mm thick, 3 × 3 mm in-plane resolution with no skip) were collected covering most of the brain, except for the anterior temporal and frontal lobes (TR = 1.5 s, TE = 29 ms, flip angle = 90°, matrix = 72 × 72). For the second version of the superior IPS localizer scans, the retinotopic mapping localizer scans, and the localizer scans for LOT, VOT, and inferior IPS, 64 interleaved axial-oblique slices taken 25 degrees toward coronal from ACPC alignment (2.3 mm isotropic voxels) were collected covering the whole brain (TR = 650 ms, TE = 34.8 ms, flip angle = 52°, matrix = 90 × 90, SMS Factor = 8). Different slice prescriptions were used here for the different localizers to be consistent with the parameters we used in previous studies. Because the localizer data were projected into the volume view and then onto individual participants’ flattened cortical surface, the exact slice prescriptions used had minimal impact on the final results. For all functional scans, T2*-weighted gradient-echo, echo-planar sequences were used, and 84 axial slices parallel to the AC-PC line (1.5 mm isotropic) were collected covering the whole brain (TR = 2 s, TE = 30 ms, flip angle = 80°, matrix = 136 × 136).

### 2.6 Data analysis

FMRI data were analyzed using FreeSurfer (https://surfer.nmr.mgh.harvard.edu), FsFast (Dale et al., 1999), and in-house Python scripts. FMRI data preprocessing included 3D motion correction, slice timing correction, and linear and quadratic trend removal. No spatial smoothing was applied. A generalized linear model (GLM) was used to estimate the responses of each voxel on every block of the experiment. The GLM analysis was run in native voxel space, using the three motion parameters as nuisance regressors. The first six TRs of each run (prior to the presentation of the first stimulus) were included as nuisance regressors to remove them from further analysis. Beta weights were estimated for each trial, where each regressor consisted of a single 8 s boxcar function convolved with the default Statistical Parametric Mapping (SPM) hemodynamic response function.

#### 2.6.1 ROI definitions

Using independent localizers, we defined ROIs based on functional and retinotopic criteria (Fig. 1B). For all ROIs, the results of the respective localizer paradigms described above were projected onto the cortical surface using Freesurfer and manually defined (details for different regions described below); ROIs were then converted to the native functional volume space of the main experiment to extract the voxels used in ROI analyses.

##### 2.6.1.1 Retinotopic ROIs

Retinotopic areas V1 through V4, V3A/B, and IPS0-4 were localized on each participant’s cortical surface by manually tracing the borders of these visual maps activated by the vertical meridian of visual stimulation (identified by locating the phase reversals in the phase-encoded mapping), following the procedure outlined inSereno et al. (2001)andSwisher et al. (2007).

##### 2.6.1.2 LOT and VOT

Following the procedure described byKourtzi and Kanwisher (2001), LOT and VOT were defined as the clusters of voxels in lateral and ventral occipitotemporal cortex, respectively, which respond more to photographs of real-world objects than to phase-scrambled versions of the same objects (*p*< .001 uncorrected). These regions correspond to the location of LO and pFs (Grill-Spector et al., 2001;Kourtzi & Kanwisher, 2001;Malach et al., 1995), but extend further into the temporal cortex in an effort to capture the continuous activations often seen extending into the ventral temporal cortex.

##### 2.6.1.3 Inferior IPS

FollowingXu and Chun (2006), the inferior IPS was defined as the cluster of voxels in the inferior portion of the IPS that responds more to arrays of four objects than to scrambled object images (*p*< .001 uncorrected).

##### 2.6.1.4 Superior IPS

FollowingTodd and Marois (2004), the superior IPS was identified in each participant as a region in the superior part of IPS that tracked each participant’s VSTM capacity using that participant’s behavioral VSTM capacity K score (Cowan, 2001), with a statistical threshold of*p*< .001 (uncorrected). We further used the Talairach coordinates reported byTodd and Marois (2004)to help us localize this ROI in each participant.

#### 2.6.2 Multivoxel pattern analysis

In a series of analyses, we applied multivoxel pattern analysis (MVPA) to characterize how object category and task are jointly represented in the visual ROIs we examined. Broadly, we both examined how strongly these variables are individually encoded in each ROI, as well as how the representation of each variable is affected by variation in the other variable. To select the voxels to include in the analysis, we initially experimented with selecting voxels based on their average response amplitude or their ability to discriminate each pair of conditions in a t-test, but since this did not reliably improve decoding performance, we included all voxels in each ROI for simplicity, and to ensure that the different analyses for each ROI were based on the same voxels. As inVaziri-Pashkam and Xu (2017), to remove response amplitude differences across stimulus conditions, trial blocks, and ROIs, beta values were z-normalized across all the voxels for each block in each ROI. A linear support vector machine (SVM) classifier (with regularization parameter*c*= 1) was then used to discriminate activity patterns associated with different stimulus conditions. Leave-one-run-out cross-validation was used for analyses involving just one task, with two adjacent runs at a time being left out for analyses involving both tasks, since each run contained only one task (see alsoVaziri-Pashkam & Xu, 2017).

In order to streamline several analyses, regions were grouped together based on their anatomical locations and the similarity of their responses to form sectors. Specifically, the results for V1, V2, V3, and V4 were averaged into a single V1–V4 sector, the results for LOT and VOT were averaged into a single LOT/VOT sector, and IPS2, IPS3, and IPS4 were averaged into a single IPS2-4 sector. These sectors were chosen in order to compare responses across the beginning of visual processing, and the end of visual processing in the ventral and dorsal streams; IPS0, IPS1, and V3/AB were not included in these sectors, since they lie at the intersection of V1–V4, LOT/VOT, and IPS2-4 and would thus not cleanly isolate any processing unique to each stream. Averaging was always performed at the final stage of each analysis in order to equate the contribution of each ROI to the final value.

In assessing statistical significance, one-tailed tests were generally used when comparing decoding with chance, and when only one direction of an effect would be meaningful (e.g., in testing for a cross-decoding drop), and two-tailed tests were used when either direction of an effect would be of interest (e.g., comparing decoding between two tasks or between two regions). Correction for multiple comparisons was applied using the Benjamini–Hochberg procedure (Benjamini & Hochberg, 1995) with false discovery rate controlled at q < 0.05. For decoding analyses performed within a single ROI or ROI sector (i.e., not involving the comparison of multiple ROIs), correction was applied within each ROI or ROI sector across all decoding analyses performed for that ROI (described below), for a total of 12 tests per ROI: (1) comparing category decoding with chance for the Oddball task, (2) comparing category decoding with chance for the Oneback task, (3) comparing category decoding between the Oddball and Oneback tasks, (4) comparing overall category decoding with chance (i.e., after averaging together category decoding for the Oddball and Oneback tasks), (5) comparing task decoding with chance, (6) comparing category and task decoding, (7) comparing category cross-decoding with chance, (8) comparing within-task category decoding to category cross-decoding, (9) testing for a category cross-decoding drop using the ratio method, (10) comparing task cross-decoding with chance, (11) comparing within-category task decoding to task cross-decoding, and (12) comparing pattern difference decoding with chance. The test for a change in each region’s category representational geometry across tasks was corrected separately from the decoding analyses, since it involved an entirely separate analysis procedure; for this analysis, correction was applied across the 12 ROIs, and separately across the three sectors. For analyses involving pairwise comparisons between sectors, correction was performed over all the pairwise comparisons performed for that analysis (e.g., for comparing the category decoding among three sectors, correction would be performed across the three pairwise tests).

##### 2.6.2.1 Category and task decoding

We performed a series of analyses to characterize how category and task are represented in the ROIs and the ROI sectors that we examined. To examine how category is represented in each ROI within each task, we computed the decoding accuracy between each possible pair of the eight categories used in the experiment (always training and testing within the same task), took the average for each participant, and ran a one-sample, one-tailed t-test comparing decoding accuracy with chance. One-tailed t-tests were used here, and in all subsequent analyses comparing decoding with chance, because only comparisons above chance were meaningful. To compare mean category decoding accuracy between the two tasks within each ROI, we used within-subjects two-tailed t-tests; two-tailed tests were used because an effect in either direction would be equally meaningful and interpretable. We then examined how task is represented in each ROI in an analogous manner. We trained a classifier to discriminate patterns drawn from trials with the same category, but different tasks, taking the average over all categories. This average was compared with chance using a one-tailed, one-sample t-test. To compare category and task decoding in each ROI, we performed two-tailed, within-subjects t-tests comparing the mean category decoding (averaged across the two tasks) with the mean task decoding.

Finally, we examined how the magnitude of category and task decoding varies between sectors. Specifically, we applied within-subjects two-tailed t-tests comparing category decoding between a V1–V4 sector, an LOT/VOT sector, and an IPS2-4 sector, with correction for multiple comparisons being applied across the three pairwise ROI contrasts. The same was done for task decoding.

##### 2.6.2.2 Characterizing nonlinear mixed selectivity coding for object category and task

**Fig. 2.**
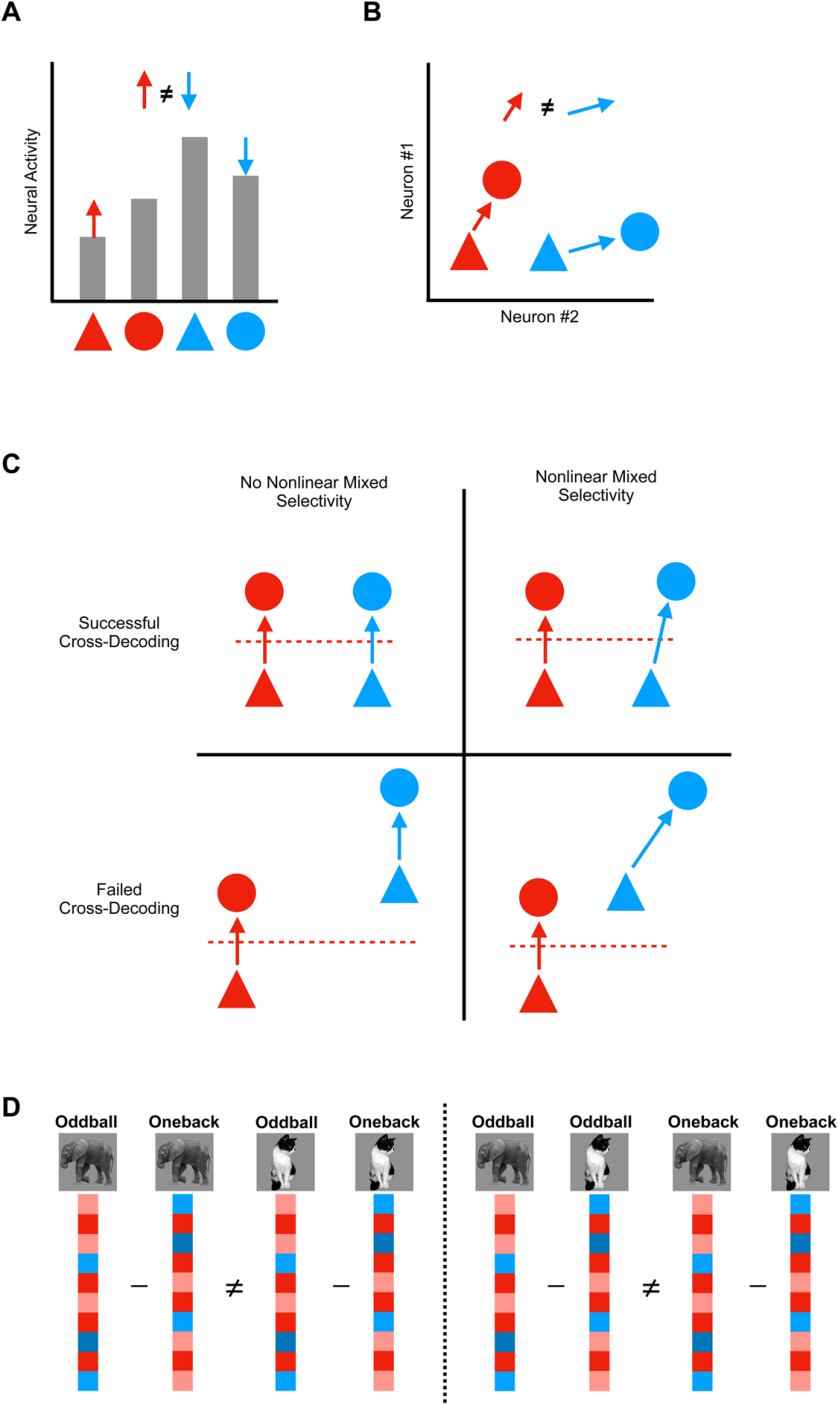
Nonlinear mixed selectivity and its relation to cross-decoding analyses. (A) Illustration of nonlinear mixed selectivity coding for a single hypothetical neuron. Each bar shows the response to a particular combination of two features (here illustrated as color and shape), with the arrows showing the response difference between the triangle and circle for each color. This neuron exhibits an interaction effect, or difference-in-differences, in its tuning profile to two properties, with the triangle-to-circle difference varying across colors. (B) Illustration of nonlinear mixed selectivity for multiple neurons. In the multivariate case, nonlinear mixed selectivity corresponds to a difference in the difference vectors between two activity patterns for different values of one variable (here, shape) across different values of another variable (here, color). (C) Relationship between nonlinear mixed selectivity and cross-decoding analyses. Dashed line is a classifier trained to discriminate between the red triangle and red circle. While nonlinear mixed selectivity can lead to a failure to cross-decode across contexts (bottom right), cross-decoding can also succeed in the presence of nonlinear mixed selectivity if the patterns remain on the correct side of the classification boundary (top right), and cross-decoding can fail in the absence of nonlinear mixed selectivity for certain geometries, such as oblique parallelogram geometries (bottom left) in which the classification boundary does not generalize properly. (D) Illustration of the pattern difference decoding analysis used in the study. To test for nonlinear mixed selectivity coding of object category and task information, the z-normalized BOLD patterns associated with presentations of the same stimulus category across different tasks were extracted, and the difference vectors between pairs of such patterns were computed. This was performed for each stimulus category, and a support vector machine classifier was then used to test whether these between-task difference vectors varied across categories (left). The same procedure was applied to test whether category-based pattern differences vary across tasks (right), and the results of these analyses were averaged, since both test for the presence of a multivariate category-by-task interaction effect.

At its core, nonlinear mixed selectivity coding is defined as an interaction effect in a neuron’s response to changes in different stimulus or task variables. At the population level, this may be reflected in a nonadditive representation of the features in the representational space (Fig. 2). Such a representation can be measured in human fMRI response patterns by training a linear classifier to decode the patterns from a pair of stimulus conditions and seeing how well it may generalize to the patterns of another pair of stimulus conditions. A significant drop in cross-decoding as compared with within-decoding (in which training and testing are performed within the patterns of the same pair of stimulus conditions) may then signal the presence of nonlinear mixed selectivity coding for the encoded features.

While an absence of nonlinear mixed selectivity can indeed accompany successful cross-decoding (Fig. 2C, top left), and the presence of nonlinear mixed selectivity can contribute to failed cross-decoding (Fig. 2C, bottom right), nonlinear mixed selectivity and a failure to cross-decode may also dissociate. For example, cross-decoding can succeed for a neural population with units exhibiting nonlinear mixed selectivity (Fig. 2C, top right) if at least some units exhibit pure selectivity (providing dimensions along which the classifier can generalize between contexts), and cross-decoding can in principle fail even for populations exhibiting no nonlinear mixed selectivity for neural populations exhibiting representational geometries that are additive but nonorthogonal (Fig. 2C, bottom left). Furthermore, a cross-decoding drop can occur when a classifier is trained in a low-noise context and tested in a high-noise context even if the patterns are otherwise identical. Cross-decoding is thus only an indirect and imperfect measure of nonlinear mixed selectivity, making it desirable to develop methods to test it directly.

To do so, here we use a technique,*pattern difference decoding*, which was first developed in a recent study to test for interactive coding of color and form in human ventral visual cortex (Taylor & Xu, 2022). It involves aggregating small and potentially heterogeneous interaction effects across voxels and testing whether fMRI pattern differences across values of one experimental variable would vary across values of another variable, thereby testing for the presence of multivariate interaction effects in a neural population. Here we use this technique to test if nonlinear mixed selectivity coding exists for representing a visual feature and its task context, allowing us to examine whether previous neurophysiology reports of such coding in association areas such as PFC and PPC may be seen with fMRI. We also compare the results of this method directly with those of the more conventional method of testing for a drop in cross-decoding when training a classifier in one context and testing it in another context. To better understand the nature of nonlinear mixed selectivity coding, using representational similarity analysis (RSA;Kriegeskorte & Kievit, 2013), we further analyzed how task may change the object representational geometry and examined the connection between nonlinear mixed selectivity coding and task-induced changes in object representational geometry.

##### 2.6.2.3 Cross-decoding analysis

Despite the caveats noted above, it is still informative to conduct cross-decoding analyses in each ROI and each ROI sector to test the extent to which the category and task decoding we observed was tolerant across variation in the other variable. To do so, for each possible pair of categories, we trained a classifier to distinguish those categories within one task, and tested the classifier on that same pair of categories in the other task. The average across both directions was taken, and the resulting values were compared with chance using a one-sample, one-tailed t-test. To test for a drop in performance when training and testing in different tasks, we performed a one-tailed, within-subjects t-test comparing the category cross-decoding accuracy with the within-task decoding accuracy (averaged across the two tasks); a one-tailed test was used since only a drop in cross-decoding, and not an increase, is interpretable. We additionally tested for a cross-decoding drop in the same three sectors (V1–V4, LOT/VOT, and IPS2-4) that were tested for the regular category and task decoding analyses.

Since brain regions can vary in their baseline decoding accuracy, in order to better compare the magnitude of the cross-decoding drop across brain regions, we computed a category cross-decoding ratio for each region. Specifically, we divided each participant’s between-task category decoding accuracy by their within-task category decoding accuracy, after subtracting .5 from both quantities (since this corresponds to chance accuracy). One-sample, one-tailed t-tests were used to compare this ratio with 1 for each ROI. This same analysis was performed for the V1–V4, LOT/VOT, and IPS2-4 sectors (averaging the ratios of the composite ROIs at the final stage), and pairwise within-subjects t-tests were used to compare the cross-decoding ratio among the three sectors. Since it is possible for decoding accuracy to vary in a nonlinear manner with the underlying dissimilarity between the two patterns being decoded, to assess the robustness of these results we also performed a version of this analysis where we first converted the original decoding accuracies to a ratio scale using the probit transform before computing the cross-decoding ratio. We note that this adjustment is only relevant for this specific analysis, since it involves subtracting the baseline accuracy and computing a ratio of two decoding accuracies.

To examine whether task was decodable across variation in object category, in each ROI and each ROI sector, we performed a cross-decoding analysis in which we trained a classifier to discriminate task for one stimulus category, and tested it on another stimulus category, taking the average over all possible pairs of categories and train–test directions. We tested both whether cross-decoding was above chance (one-tailed t-tests) and whether there was a drop in cross-decoding compared with within-decoding (one-tailed t-tests). The same analyses were applied to the V1–V4, LOT/VOT, and IPS2-4 sectors, as before. Since no significant cross-decoding drop for task was found in any ROI, we did not compute a cross-decoding ratio as we did in the case of category cross-decoding.

##### 2.6.2.4 Pattern difference decoding

Next, we examined the extent to which the joint code for category and task in each ROI exhibits nonlinear mixed selectivity—that is, an interaction effect. Since single voxels are noisy, and since different voxels could in principle exhibit different interaction effects (e.g., different voxels might prefer different category–task combinations), we applied an analysis,*Pattern Difference Decoding*, that uses a support vector machine classifier to test for the presence of distributed multivariate interaction effects (Fig. 2D). Specifically, we extracted the z-normalized activity patterns for a pair of blocks that had the same task (e.g., Oddball), but different categories, and took the difference vector between these patterns. We then did the same for the other task (e.g., Oneback), and used a support vector machine to test whether these difference vectors were discriminable from each other—that is, whether a systematic “difference in differences” is present. Successful decoding requires that the difference vector between the patterns corresponding to two different categories is relatively stable within a task, but different between tasks, such that a decoder is capable of discriminating the between-category difference vectors from the two tasks. This procedure was performed over all possible pairs of categories. The complementary analysis of discriminating task difference vectors across categories was also performed, and the results were averaged, since both directions of the analysis test for the presence of an interaction effect, and since decoding accuracy was highly correlated across the two dimensions. The resulting mean decoding accuracies were then compared with chance using one-sample, one-tailed t-tests. This was done separately for each ROI and each ROI sector. To test for differences in nonlinear mixed selectivity among ROIs, we performed pairwise within-subjects t-tests between the V1–V4, LOT/VOT, and IPS2-4 sectors.

Since measuring the strength of nonlinear mixed selectivity across the human visual system was a primary goal of the study, we performed two additional analyses to quantify the robustness of the pattern difference decoding results. First, we performed a nonparametric permutation test analysis to assess how many individual participants exhibited significant pattern difference decoding for each ROI and sector. Specifically, we shuffled the training labels for the pattern difference vectors immediately prior to using them to train the support vector machine, did this one thousand times in order to generate a null distribution reflecting the distribution of decoding accuracies under the hypothesis of no significant pattern difference decoding, and computed a*p*-value for each participant by computing the proportion of permuted iterations with a higher accuracy than the nonpermuted data. We then tallied how many participants had a*p*-value less than .05, after correcting for multiple comparisons across the 13 participants. Second, in order to better quantify the strength of the evidence for significant pattern difference decoding in each ROI, we employed the variational Bayesian mixed effects analysis developed byBrodersen et al. (2013)in order to compute the posterior odds of above-chance pattern difference decoding (i.e.,*p*(accuracy > .5) /*p*(accuracy < .5)).

##### 2.6.2.5 Representational similarity analysis

To understand how the presence of nonlinear mixed selectivity coding of category and task may correspond to changes in the object representational structure across different brain regions, we conducted representational similarity analysis (RSA,Kriegeskorte & Kievit, 2013).

First, to visualize the overall representational structure of category and task in several example ROIs, we conducted a multidimensional scaling (MDS) analysis. To do so, we computed the pairwise classification accuracy for every possible pair of the 16 total block conditions (8 categories by 2 tasks) to construct a representational dissimilarity matrix (RDM). We next converted the averaged RDM (across participants) into a valid RDM for multidimensional scaling by subtracting .5 from each classification accuracy (since .5 is chance performance) and by setting any resulting negative values to zero. The resulting values were averaged across regions to obtain average RDMs for the V1–V4, LOT/VOT, and IPS2-4 sectors, after which MDS was applied to the resulting pairwise dissimilarities among the 16 conditions.

We then performed a “second-order” MDS analysis to characterize the similarity of the representational space across different regions and tasks. To do this, we computed the mean decoding accuracy across participants for all pairwise comparisons between categories within each ROI and task, yielding a category similarity vector for each task and each ROI. We then computed the Pearson correlation for every possible pair of these category similarity vectors, subtracted these values from 1 to convert these correlations into a distance metric, and visualized the resulting dissimilarity structure using MDS.

While the cross-decoding and pattern difference decoding analyses test for the presence of category–task interaction effects, they do not reveal how this interaction effect is reflected in each region’s representational geometry. For instance, an interaction effect could arise in a case where a brain region’s category representational geometry is rotated between tasks while keeping the relative distances between the categories constant, or it could arise from a case where these distances themselves are altered between tasks. Whereas the former does not involve a change in the representational content as the relative similarities among the categories are preserved, the latter changes the content of the representation. To arbitrate between these possibilities, we tested whether the category representational geometry significantly varies between tasks for each region. To account for measurement noise across different brain regions to allow us to make valid cross-region comparisons, we obtained a noise ceiling or reliability measure for each region and took this into account when we compared results across regions. Specifically, from the pairwise category decoding accuracy within each task, we constructed a category RDM and took the off-diagonal values to create a category similarity vector for each participant for that task. We then correlated each participant’s category similarity vector (without subtracting .5 and truncating to zero, since this was only done to allow the MDS embeddings to be computed) with the average category similarity vector across the remaining participants both for the same task (Oddball to Oddball, Oneback to Oneback), and for the opposite task (Oddball to Oneback, Oneback to Oddball), and averaged across the two within-task and the two between-task correlations. The averaged within-task RDM correlation across participants in each ROI provides a correlation noise ceiling or reliability measure for that region (i.e., the overall RDM consistency across participants given the existence of measurement noise; seeKhaligh-Razavi & Kriegeskorte, 2014). To obtain a noise-ceiling or reliability-corrected measure of how each brain region’s category representational geometry is modulated by task, we divided each participant’s between-task RDM correlation by the mean within-task RDM correlation across all participants to obtain a reliability-corrected between-task RDM correlation. FollowingXu and Vaziri-Pashkam (2021), we further squared this ratio so it reflects the proportion of variance that the between-task RDM can explain for the within-task RDM. A value of 1 indicates no RDM changes between tasks, whereas any value lower than 1 indicates a difference in RDMs between the two tasks. The resulting value was compared with 1 using one-sample, one-tailed t-tests both for each individual ROI, and for the V1–V4, LOT/VOT, and IPS2-4 sectors, with correction for multiple comparisons being applied separately across all the individual ROIs (i.e., across 12 tests) or across all the sectors (i.e., across 3 tests). One-tailed tests were used since only a drop in RDM correlation for between-tasks relative to within-tasks is meaningful. Finally, within-subjects, two-tailed t-tests were used to assess whether the magnitude of the task-induced representational geometry change varied among the three sectors, corrected for multiple comparisons across the three pairwise sector contrasts.

To better understand how the magnitudes of the task-induced representational geometry change relate to the interaction effects measured using pattern difference decoding, for sectors showing a significant change in category representational geometry based on task, we correlated the magnitude of this change with the magnitude of pattern difference decoding within each participant and tested the significance of this correlation (two-tailed). Since pattern difference decoding is a within-participant metric (i.e., without taking into account the performance of other participants), in order to make the task-induced representational geometry change measure comparable and be a likewise within-participant metric, instead of obtaining this measure at the group level as described earlier in which the measure depended on the performance of other participants, here we simply computed each participant’s category RDM correlation between tasks directly.

## 3 Results

A primary goal of the study was to noninvasively test for the presence of nonlinear mixed selectivity coding of object and task information in the human visual system, since all prior studies testing for nonlinear mixed selectivity of stimulus and cognitive variables have required invasive neural recording techniques. Additionally, we sought to understand how nonlinear mixed selectivity for object and task affects the object representational geometry—specifically, whether it simply reflects a shift/rotation of the geometry without changing the structure of the geometry or whether it actually corresponds to a structural change in the geometry. To do so, we examined how category and task are jointly represented across early, ventral, and dorsal ROIs by having participants perform one of two tasks on stimuli from eight different categories. The task could either be an Oddball task, in which the participant was to respond if the category of a stimulus did not match that of the surrounding block, or a Oneback task, in which the participant was to respond if exactly the same stimulus was repeated twice in a row. These tasks were chosen so as to equate spatial, object-based, and feature-based attention, thereby allowing us to test which brain regions still exhibit task-modulated visual representations even when these forms of attention are equated. Using fMRI MVPA, we tested both how category and task are individually represented, and how the representation of each variable is affected by variation in the other. To better understand the nature of nonlinear mixed selectivity coding, using RSA (Kriegeskorte & Kievit, 2013) we further analyzed how task may change the object representational geometry and examined the connection between nonlinear mixed selectivity coding and task-induced changes in object representational geometry.

### 3.1 Representation of category and task

**Fig. 3.**
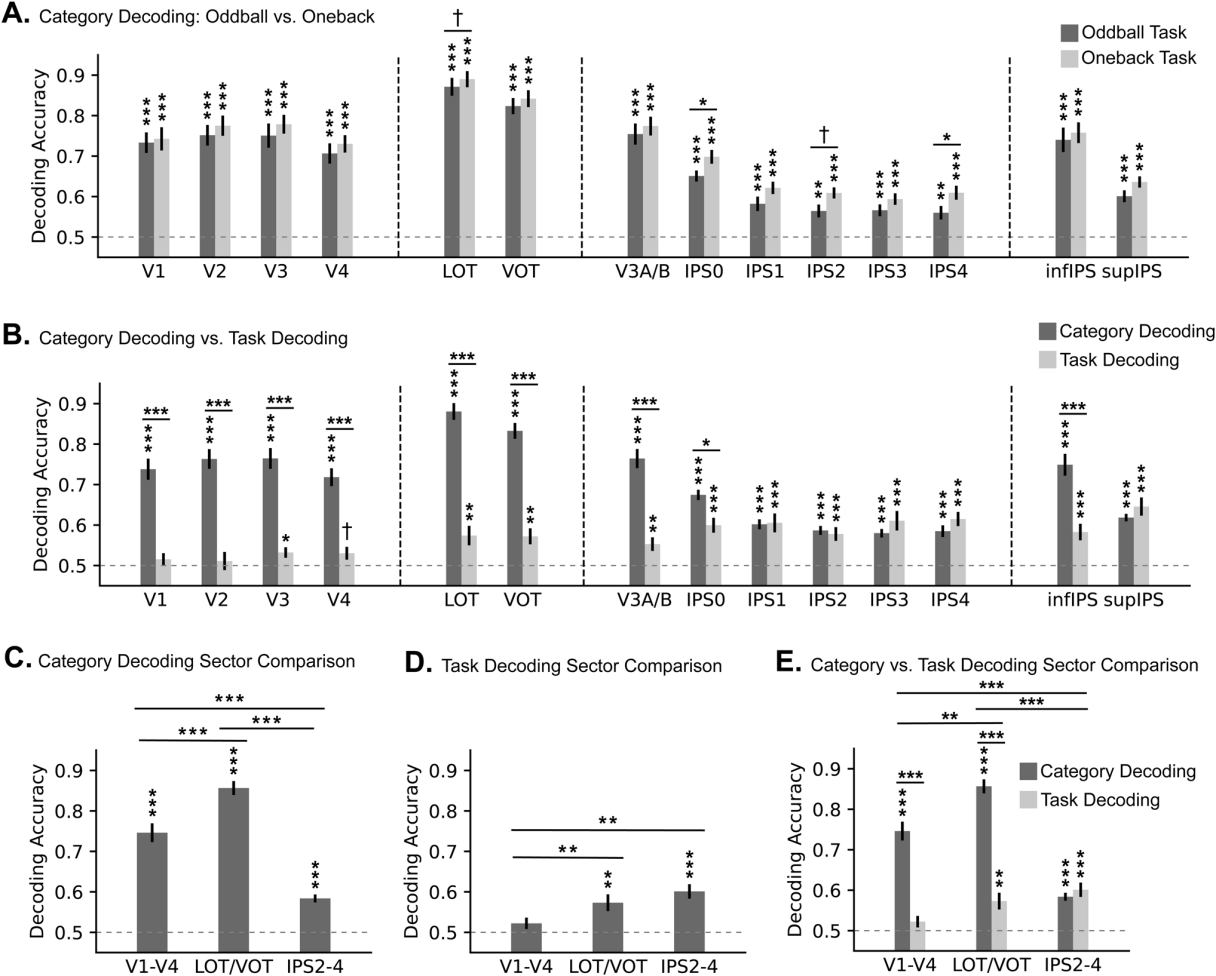
Coding of category and task. (A) Category decoding accuracy separately for the Oddball and Oneback tasks. (B) Direct comparison of category and task decoding for each ROI. (C) Sector-wise comparison of category decoding for V1–V4, LOT/VOT, and IPS2-4. (D) Sector-wise comparison of task decoding for V1–V4, LOT/VOT, and IPS2-4. (E) Sector-wise comparison of category–task decoding differences for V1–V4, LOT/VOT, and IPS2-4.^†^*p*< .1; *p< .05; **p< .01; ***p< .001 for t-tests testing for above chance (>.5) decoding (all one-sample t-tests, one-tailed, and corrected for multiple comparisons across all analyses performed for each ROI), and for within-subjects t-tests comparing different conditions either within an ROI or between ROIs (corrected for multiple comparisons across all pairs of ROIs that were compared).

We first used MVPA to document the strength of category and task decoding in each ROI (Fig. 3). Prior work has found dorsal visual representations to be strongly modulated by task (Vaziri-Pashkam & Xu, 2017;Xu & Vaziri-Pashkam, 2019), but these studies compared pairs of tasks with different attentional demands, making it important to establish whether these task-induced modulations persist even for tasks with similar attentional demands. To do so, we first examined the strength of category decoding separately in the Oddball and Oneback tasks. All ROIs exhibited significantly above-chance decoding of object category in both tasks (see the asterisks marking the significance levels inFig. 3; one-tailed and corrected). Several ROIs showed higher category decoding in the Oneback task than the Oddball task, with others showing no difference, and none showing the opposite effect (see the asterisks marking the significance levels inFig. 3; one-tailed and corrected). One possible explanation for this is that the Oneback task requires more sustained stimulus retention, which could have boosted the signal-to-noise ratio and enhanced category-diagnostic visual information.

To compare across the ROIs, to maximize the contrast, and to streamline analysis, we averaged the decoding performance of several ROIs into three sectors to broadly compare the strength of stimulus category representation across the early, ventral, and dorsal visual pathways. Specifically, we created a sector consisting of early visual areas V1–V4, a sector consisting of ventral object areas LOT and VOT, and a sector consisting of dorsal areas IPS2-4. LOT/VOT showed significantly higher category decoding than V1–V4, which in turn showed significantly higher decoding than IPS2-4 (see the asterisks marking the significance levels inFig. 3C; two-tailed and corrected).

Next, we examined how task is represented in these regions. We trained a classifier to decode task within each of the eight stimulus categories, and averaged the resulting accuracies. Task was either not decodable or weakly decodable in early visual regions, modestly though significantly decodable in LOT and VOT, and reliably decodable across the dorsal stream regions we examined (see the asterisks marking the significance levels inFig. 3B; one-tailed and corrected). We also contrasted task and category decoding and found significantly lower task than category decoding in early visual areas and ventral areas, but similar levels of decoding in dorsal areas (see the asterisks marking the significance levels inFig. 3B and E; two-tailed and corrected). Pairwise comparisons among V1–V4, LOT/VOT, and IPS2-4 showed significantly higher decoding of task for LOT/VOT and IPS2-4 than for V1–V4, with a greater category and task decoding difference in V1–V4 and LOT/VOT than in IPS2-4, and a greater difference in LOT/VOT than in V1–V4 (see the asterisks marking the significance levels inFig. 3D and E; two-tailed and corrected).

### 3.2 Testing for nonlinear mixed selectivity for category and task

**Fig. 4.**
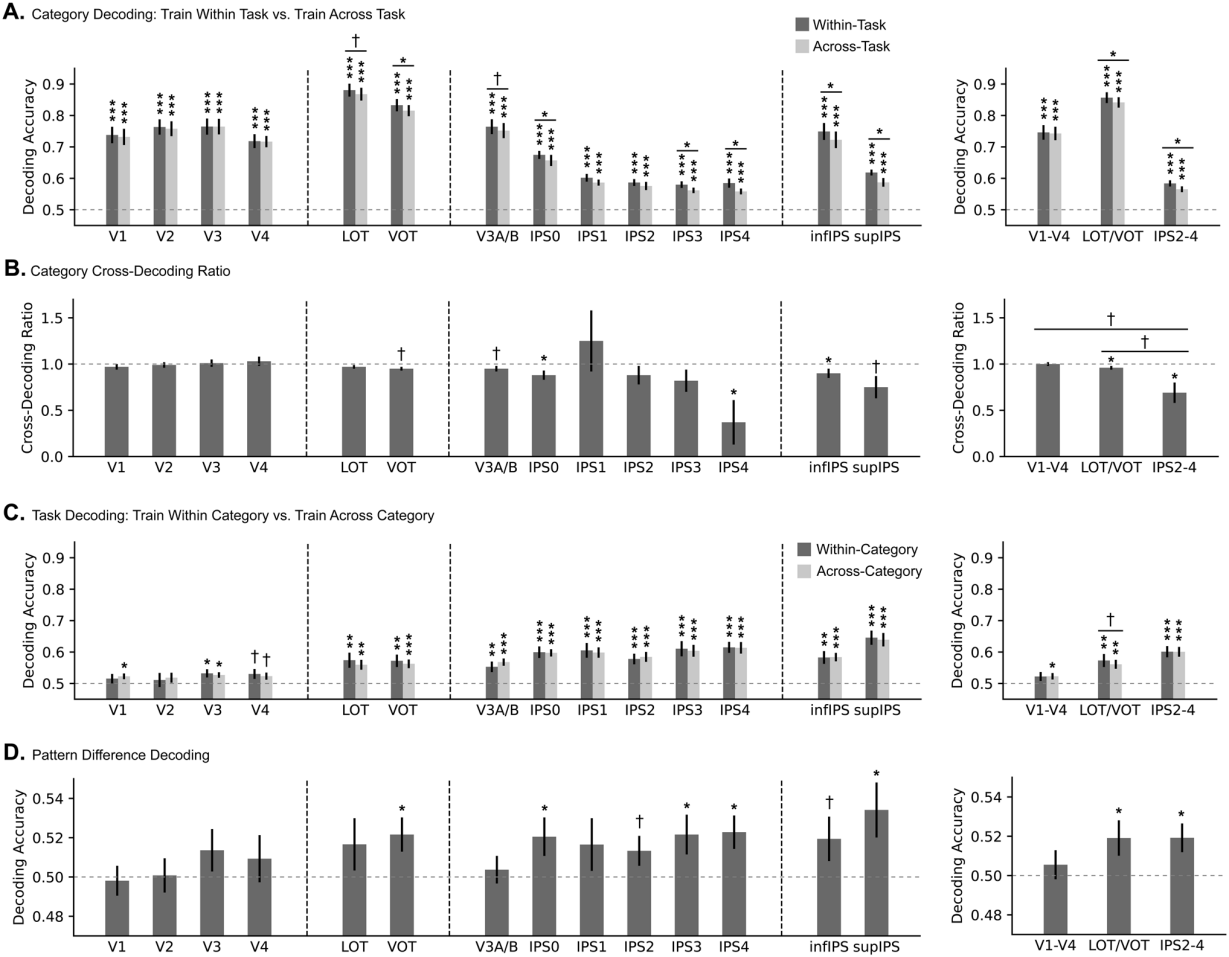
Results of cross-decoding and pattern difference decoding analyses testing for nonlinear mixed selectivity coding. (A) Category decoding when a classifier is trained and tested to decode category within the same task, versus when it is trained on one task and tested on the other task. Right panel shows within-task and between-task category decoding for the three sectors. (B) Category cross-decoding ratio, computed by dividing the between-task cross-decoding accuracy by the within-task decoding accuracy. Right panel shows values for three sectors. (C) Task decoding when a classifier is trained and tested to decode task within the same stimulus category, versus when it is trained on one category and tested on another category. Right panel shows values for three sectors. (D) Results of pattern difference decoding analysis testing for interactive coding of category and task. Above-chance decoding is indicative of significant interactive coding of stimulus category and task. Right panel shows average values for three sectors.^†^*p*< .1; *p< .05; **p< .01; ***p< .001 for one-sample, one-tailed t-tests testing for above-chance (>.5) decoding, and for within-subjects t-tests comparing different conditions either within an ROI or between ROIs (corrected for multiple comparisons across all pairs of ROIs that were compared).

Having established that both category and task are decodable in PPC and higher OTC regions, we turn to the critical question of whether these two variables exhibit nonlinear mixed selectivity in their neural tuning to these variables (Fig. 4). We tested for this using two different analyses: testing for a cross-decoding drop when training a classifier on one value of a given variable (category or task) and testing on another value, and via an approach called*Pattern Difference Decoding*that directly tests for the presence of multivoxel interaction effects.

When training a classifier to decode category in one task and then testing it on the other task, we found that VOT, IPS0, IPS3, IPS4, infIPS, supIPS, the LOT/VOT sector, and the IPS2-4 sector all showed a significant cross-decoding drop, with LOT and V3A/B showing trends, indicating a task modulation of the category representations encoded by these regions (Fig. 4A; see the asterisks marking the significance levels; one tailed and corrected).

To compare the magnitude of the cross-decoding drop among ROIs and to account for differences in the overall category decoding strength in an area, followingXu and Vaziri-Pashkam (2022), we additionally computed a cross-decoding ratio for each ROI and sector (Fig. 4B). This was done by first subtracting .5 from the accuracy values, and then computing the ratio of the within- and cross-decoding accuracies (such that a ratio less than 1 indicates a cross-decoding drop). With this measure, we found that the cross-decoding ratio was no different from 1 in V1–V4, but significantly lower than 1 in both LOT/VOT and IPS2-4 (see the asterisks marking the significance levels inFigure 4B; one-tailed and corrected). Moreover, the cross-decoding ratio was marginally smaller in IPS2-4 than in either V1–V4 (*t*(*12)*= 2.63,*p*= .06) or LOT/VOT (*t(12)*= 2.28,*p*= .06), after correction for multiple comparison across the three pairwise tests comparing the cross-decoding ratio between sectors. We obtained similar results after first converting the decoding accuracies to a ratio scale using the probit transform before computing the cross-decoding ratio, with IPS2-4 showing a marginally smaller ratio than V1–V4 (*t*(*12)*= 2.60,*p*= .07) or LOT/VOT (*t(12)*= 2.09,*p *= .087), and LOT/VOT additionally trending toward showing a smaller ratio than V1–V4 (*t(12)*= 1.82,*p*= .093). These results suggest that when the overall category decoding strength is taken into account, there appears to be a larger cross-decoding drop in IPS2-4 than in the other two sectors.

We then performed the complementary analysis of examining whether training a classifier to decode task on one category and testing on another category exhibited a drop in decoding relative to training and testing within the same category. Here, no regions showed a significant drop, with the exception of a trending difference for the LOT/VOT sector (Fig. 4C).

While a drop in cross-decoding can sometimes indicate the presence of an interaction effect (Fig. 2C, bottom right panel), these two measures can also dissociate: an interaction effect can be present even with no drop in cross-decoding (Fig. 2C, top right panel), and a drop in cross-decoding can occur even when no interaction effect is present (Fig. 2C, bottom left panel). Thus, in order to directly test for the presence of a category/task interaction effect in each ROI, we applied a technique called*Pattern Difference Decoding*that involves training a classifier to test whether differences in activity patterns between trials of different categories in the same task vary across tasks, and vice versa (seeSection 2for more details). Above-chance pattern difference decoding is indicative of the presence of a multivariate “difference in differences,” or interaction effect, in the joint coding of object category and task. First, in order to verify that the two directions of the analysis—decoding category differences across task and decoding task differences across categories—could be sensibly averaged, we correlated the accuracies for the two directions across participants within each ROI. All ROIs showed a high correlation between the two directions (all*r*s > .69; average*r*of .85), so decoding accuracy was averaged across the two directions to yield the final decoding accuracy for each ROI. Several dorsal stream regions—specifically, IPS0, IPS3, IPS4, and supIPS—and one ventral stream region, VOT, exhibited significant pattern difference decoding, with IPS2 showing a trend (Fig. 4D; see the asterisks marking the significance levels; one tailed and corrected). Significant pattern difference decoding was also found in the LOT/VOT and IPS2-4 sectors, but not in the V1–V4 sector, indicating the existence of nonlinear mixed selectivity tuning for task and category in these sectors. Pairwise comparisons showed no significant differences in pattern difference decoding among the V1–V4, LOT/VOT, and IPS2-4 sectors (*t*s < 1.3,*p*s > .21; two-tailed and corrected).

To better characterize the robustness of pattern difference decoding across the ROIs we examined, we performed two additional analyses (Table 1). First, we performed a nonparametric permutation test in order to assess the significance of pattern difference decoding for each individual participant (corrected for multiple comparisons within each ROI across the 13 participants). Superior IPS, the LOT/VOT sector, and the IPS2-4 sector exhibited significant pattern difference decoding in more than half of the participants, and V3, VOT, IPS0, and IPS1 exhibited significant pattern difference decoding in six or more participants. Second, we performed a variational Bayesian mixed effects analysis (Brodersen et al., 2013) in order to compute the posterior odds of above-chance pattern difference decoding. VOT, IPS0, IPS3, IPS4, supIPS, the LOT/VOT sector, and the IPS2-4 sector all had a posterior odds ratio exceeding 3, indicative of moderate evidence of above-chance pattern difference decoding, with supIPS showing the highest posterior odds (6.92) of any of the ROIs we examined. Of the regions showing no significant pattern difference decoding in the main analysis, LOT, V3, V4, and IPS1 show a posterior odds greater than 1.5, indicative of modest evidence for above-chance decoding, whereas V1, V2, V4, and V3A/B have a posterior odds of approximately 1, suggestive of no evidence for significant pattern difference decoding. We thus find moderately strong evidence for above-chance pattern difference decoding in several regions, with these regions showing significant effects in a large proportion of individual participants, and moderately strong posterior odds in favor of above-chance decoding.

**Table 1.** Quantifying the robustness of pattern difference decoding.

Region/sector	# Significant participants from permutation test	Posterior odds ratio
V1	0	0.89
V2	0	1.03
V3	6	2.15
V4	4	1.66
LOT	5	2.48
VOT	6	3.57
V3A/B	1	1.22
IPS0	6	3.27
IPS1	6	2.45
IPS2	3	2.15
IPS3	5	3.48
IPS4	4	3.81
infIPS	5	2.99
supIPS	8	6.92
V1–V4	5	1.32
LOT/VOT	7	3.00
IPS2-4	8	3.07

Middle column gives the number of individual participants showing significant pattern difference decoding (permutation test; corrected for multiple comparisons across the 13 participants). Right column gives Bayesian posterior odds ratio*p*(accuracy > .5)/(*p*(accuracy < .5).

Results from pattern difference decoding were thus in strong agreement with those from the category cross-decoding drop analysis: regions that show significant pattern difference decoding also tended to show a significant category cross-decoding drop. In fact, significant correlations between the magnitude of the cross-decoding drop and that of pattern difference decoding across participants were found in all the ROIs examined (*r*s > .59,*p*s < .04). However, similar results were not obtained when decoding task across variation in object category (seeSection 4). Overall, we obtained convergent evidence showing for the first time the existence of nonlinear mixed selectivity coding for category and task in PPC and VOT using fMRI MVPA.

### 3.3 Representational similarity analysis (RSA)

**Fig. 5.**
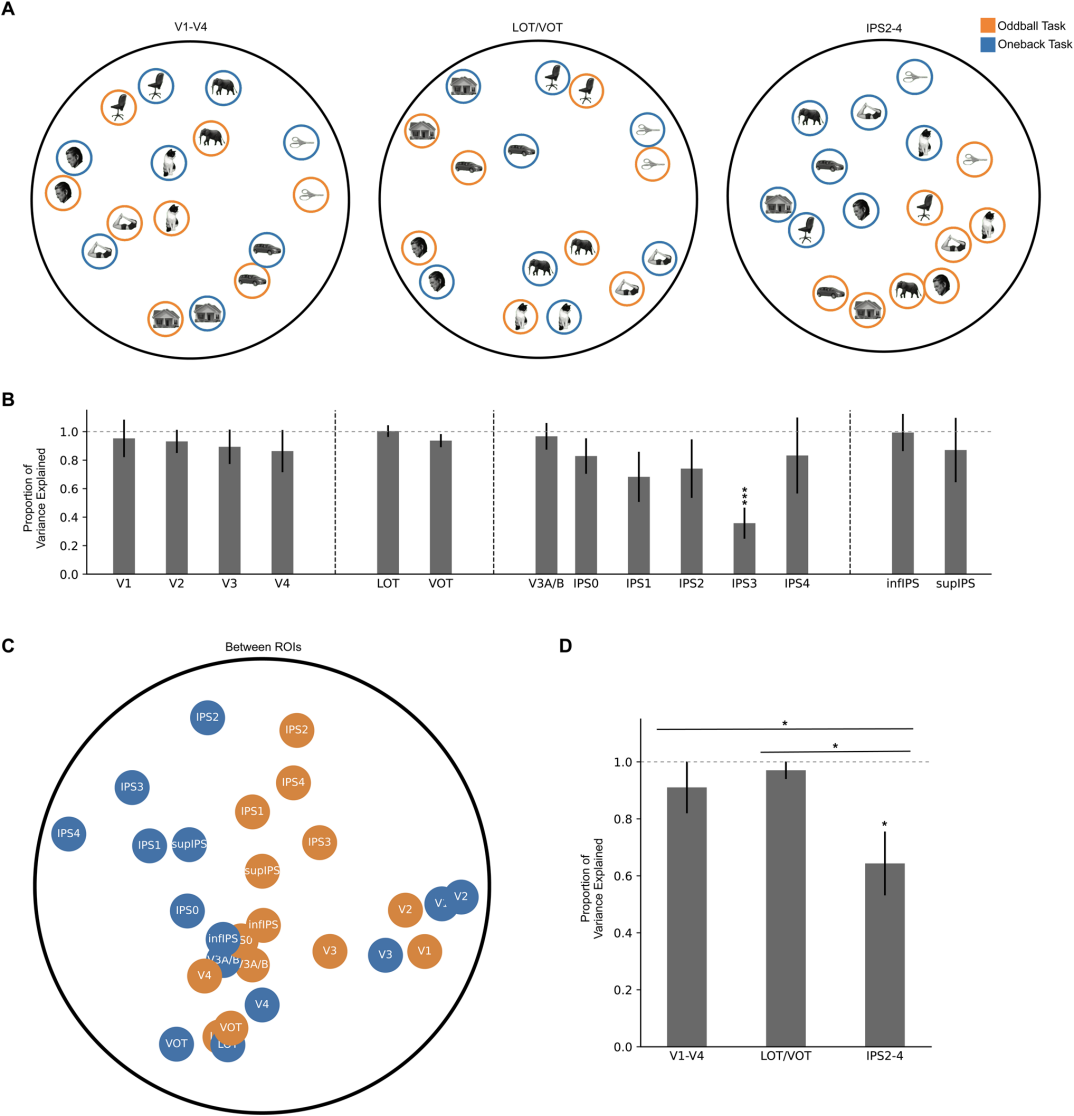
Multidimensional scaling analyses showing representational geometry within regions, how geometry varies across regions, and how geometry is modulated by task. (A) The pairwise classification accuracies from each category–task combination were computed, and multidimensional scaling was applied to project the resulting dissimilarity structure into two dimensions. Each category is represented by the example icon, and task is depicted by the color of the ring around the icon. Whereas the representational structure of V1–V4 and LOT/VOT is primarily driven by stimulus category, in IPS2-4 the task plays a much stronger role in shaping the representational geometry of the sector. (B) Proportion of within-in task RDM variance explained by between-task RDM in individual brain regions. The category representational geometry was significantly more similar within-task than between-task for IPS3. (C) Second-order multidimensional scaling analysis comparing the category and task representational geometry across brain regions. The category similarity structures within each task and ROI were computed, the resulting similarity structures were compared across ROIs, and the resulting second-order similarities were projected onto two dimensions using MDS to characterize how representational geometry varies based on brain region and task. The dorsal stream brain regions vary along an axis oblique to the axis spanned from the early visual regions through the ventral stream. While ventral regions largely overlap across the two tasks, dorsal regions appear to diverge across tasks. (D) Proportion of within-in task RDM variance explained by between-task RDM averaged across sectors. The category representational geometry was significantly more similar within-task than between-task for the IPS2-4 sector, with a significantly greater modulation in IPS2-4 than in the V1–V4 and LOT/VOT sectors.^†^*p*< .1; *p< .05; **p< .01 for within-subjects t-tests testing for a drop in category representational geometry correlation between tasks (one-tailed, corrected for multiple comparisons across the 14 regions for B, and for the three sectors in D), and for within-subjects t-tests testing for differences in the magnitude of this drop between sectors in D (two-tailed, corrected for multiple comparisons across the three pairwise tests).

In order to examine whether the nonlinear mixed selectivity we observed reflects a mere rotation of representational geometry versus an actual reshaping of the profile of dissimilarities among different categories (indicative of a change in representational content), we used RSA (Kriegeskorte & Kievit, 2013) to understand how category, task, and their interaction modulate the representations in each region (Fig. 5).

We first examined object representational geometry in individual ROIs. This was done by obtaining pairwise decoding for all the conditions (8 categories x 2 tasks) to construct an object-task-wise representational dissimilarity matrix (RDM) including both category and task (seeSection 2). We then projected this RDM onto a 2D space using MDS (Fig. 5A). As inVaziri-Pashkam and Xu (2017)andVaziri-Pashkam and Xu (2019), we examined the projection in representative sectors spanning early visual, ventral visual, and dorsal areas V1–V4, LOT/VOT, and IPS2-4. Whereas in V1–V4 and LOT/VOT the representational structure is primarily driven by object category, with a much weaker secondary representation of task, in IPS2-4 both category and task play a dominant role in shaping the object representational geometry of the region, similar to the results reported inVaziri-Pashkam and Xu (2017). Task is thus still prominently represented in PPC even when the overall attended visual features are equated between tasks.

FollowingXu and Vaziri-Pashkam (2019), to characterize how representational geometry varies across different regions and tasks, we additionally performed a “second-order” MDS analysis in which we first extracted the RDM for each ROI in each task. We then correlated all the resulting RDMs to form a region-task-wise RDM and visualized the resulting similarity structure using MDS (seeSection 2). A relatively linear trajectory from V1 to LOT and VOT is evident, along with an oblique, intersecting trajectory following the dorsal visual pathway (Fig. 5C). This replicates the two-pathway structure first reported byVaziri-Pashkam and Xu (2019; see alsoXu & Vaziri-Pashkam, 2019;Xu, 2023) and shows that the object category representational geometry for the eight object categories differs among the ventral and dorsal regions. Interestingly, the regions in the dorsal pathway appear to diverge for the two tasks, especially for the higher PPC regions, indicating that the object representational geometry differs for the two tasks in PPC, despite matching the attentional demands of the two tasks. Such a separation is less evident for the regions in the ventral pathway.

To quantify these observations, we tested whether each brain region’s category representational geometry is more correlated within tasks than between tasks. Specifically, we correlated the RDM of a given participant in one task with the averaged RDMs from all other participants either in the same (within-task) or the other task (between-task) and rotated this procedure across all the participants. The averaged within-task RDM correlation across participants in each ROI provides a correlation noise ceiling or reliability measure for that region (i.e., the overall RDM consistency across participants given the existence of measurement noise; seeKhaligh-Razavi & Kriegeskorte, 2014). To account for reliability differences among ROIs when comparing how strongly task modulates their category representational geometry, we then divided each participant’s between-task correlation by the mean within-task correlation across all participants to obtain a reliability-corrected between-task correlation. FollowingXu and Vaziri-Pashkam (2021), we further squared this correlation so it reflects the proportion of variance that the between-task RDM can explain for the within-task RDM. A value of 1 would indicate no RDM change between tasks, whereas any value lower than 1 would indicate a difference in RDMs between the two tasks. To increase power, as in the previous analyses, we additionally performed this analysis on the V1–V4, LOT/VOT, and IPS2-4 sectors. Both IPS3 and the IPS2-4 sectors showed a significant change in RDMs between tasks (Fig. 5B, D). However, a similar change was not found in early visual areas V1–V4 or higher visual areas LOT/VOT. Furthermore, this change was significantly larger in the IPS2-4 sector than in either the V1–V4 or the LOT/VOT sector (*p*s < .05,*t*s > 2.47; two-tailed within-subjects t-tests, corrected for multiple comparisons across the three pairwise contrasts). Thus, higher level dorsal regions exhibit a significant task-modulated shift in category representational geometry despite the matched attentional demands between the two tasks, while early visual and ventral regions do not.

Since IPS2-4 exhibited both significant pattern difference decoding and a significant drop in category geometry correlation between tasks, we tested for a correlation between the magnitude of the pattern difference decoding accuracy in each participant and the corresponding magnitude in RDM correlation between tasks. Across participants, these quantities exhibited a significantly negative correlation (*r*= -.58,*p*< .05; two-tailed), indicating that the higher the pattern difference decoding, the less similar the RDMs were between the two tasks. Thus, the interaction effect we observed using pattern difference decoding is strongly linked to the magnitude of representational content change between the two tasks.

Overall, these results show that while nonlinear mixed decoding for category and task was found in both LOT/VOT and IPS2-4, their effects on category representation differ: while such decoding in LOT/VOT appears to correspond to a shift/rotation in the representational geometry without changing the content of representation (i.e., the relative similarities among the categories), nonlinear mixed decoding in IPS2-4 reflects a change in the representational content and not merely a shift/rotation of the representational geometry.

## 4 Discussion

Given the significant role nonlinear mixed selectivity tuning may play in information representation in the primate brain (Bernardi et al., 2020;Fusi et al., 2016;Rigotti et al., 2013), the primary goal of the present study was to test the feasibility of measuring such coding in the human brain noninvasively using fMRI pattern decoding. An added benefit of fMRI is that it allows multiple brain areas to be examined and compared within the same participants, compared with the limited spatial coverage of neurophysiological studies. To do so, we examined the joint representation of category and task information across human early, ventral stream, and dorsal stream ROIs. Given that the mere presence of nonlinear mixed selectivity coding of objects and tasks does not directly reveal the nature of such coding, an equally important goal was understanding how nonlinear mixed selectivity coding of objects and tasks may relate to changes in how objects are represented across the different tasks.

In the present study, participants were presented with stimuli from eight different categories, and performed either an Oddball task or a Oneback task. These tasks were chosen so as to equate spatial, object-based, and feature-based attention, in order to test whether task modulations of visual representations still occur when the inputs to visual processing are kept constant between the two tasks, with only the subsequent cognitive operations varying. We found nonlinear mixed selectivity tuning in fMRI response patterns in both human ventral and dorsal areas and demonstrate the feasibility of using fMRI MVPA to document this coding mechanism noninvasively in the human brain. Importantly, we showed that while such coding in ventral areas corresponds to a rotation or shift in the object representational geometry without changing the representational content (i.e., with the relative similarity among the categories preserved), nonlinear mixed selectivity coding in dorsal areas is linked to a change in representational content, with the magnitude of such coding correlated with the magnitude of representational content change across participants.

Before testing for nonlinear mixed selectivity coding of category and task information, we first tested whether category and task information alone could be robustly decoded in each ROI. Past work has reported stronger object category representation in human ventral than in dorsal regions, and the reverse pattern of results for task representation, with stronger task representation in human dorsal than in ventral regions (Vaziri-Pashkam & Xu, 2017). However, given that participants attended to different features of an object in the different tasks, task decoding was confounded with attentional demands, making it unclear whether task decoding still exists when the overall attended visual features are equated between tasks. ReplicatingVaziri-Pashkam and Xu (2017), we found significant category decoding across all regions examined, with decoding being higher in ventral than in early visual and dorsal regions, and higher in early visual than in dorsal regions. Importantly, task could be significantly decoded in every dorsal stream region we examined, as well as both ventral stream regions and several early visual regions. That said, while category decoding was much stronger than task decoding in the early visual and ventral regions, dorsal regions tended to show more similar levels of category and task decoding, consistent with a relatively stronger contribution of task to the representations in these regions, in line with past work (Vaziri-Pashkam & Xu, 2017). The fact that task is decodable despite matching space-, object-, and feature-based attentional demands of the two tasks suggests that task representation in PPC can reflect not just*which*visual information is selected, but also the subsequent operations performed upon that information.

Having established the presence of object category and task representation in both ventral and dorsal regions, we tested for the presence of nonlinear mixed selectivity (i.e., interactive) coding of category and task using a*pattern difference decoding*analysis that tests whether neural pattern differences based on category vary across task and vice versa. Consistent with prior neurophysiological findings, we found that multiple PPC regions exhibited significant pattern difference decoding, indicating the presence of nonlinear mixed selectivity tuning for category and task information. Notably, this implies that between-category pattern differences were sufficiently reliable within a task, and sufficiently different across tasks, to allow for successful decoding, indicative of a task-specific multivariate mapping between categories (or equivalently, a category-specific multivariate mapping between tasks). We thus show for the first time the feasibility of using fMRI MVPA to document nonlinear mixed selectivity tuning to sensory and cognitive variables noninvasively in the human brain, suggesting that not only do neurons exhibiting nonlinear mixed selectivity exist in these brain regions, but also that their tuning exhibits spatial clustering at a scale visible to fMRI. While it is theoretically possible that a population of neurons exhibiting purely linear underlying tuning could give rise to an observed nonlinear effect at the level of the BOLD response due to neurovascular coupling dynamics, several results speak against this interpretation: for example, V3, LOT, V3A/B, and IPS1 all show significant category and task decoding, but no significant pattern difference decoding.

Notably, the interactive coding we observed was of a much lower magnitude than the coding of category and task, and totally absent in most brain regions we examined. Further neurophysiological studies would help clarify whether this is due to genuinely weak nonlinear mixed selectivity tuning in the underlying neural populations, or whether it may be due to neurons with different tuning preferences canceling out in the BOLD signal, reducing the observed magnitude of the interaction effect. We additionally note that the steps taken to match the attentional demands of the two tasks may have decreased the decoding accuracy by removing the contribution of these potential confounds (although it increases our confidence that the decoding we observed reflects the downstream cognitive operations that varied between the two tasks, and not merely differences in attentional allocation).

Besides the pattern difference analysis, we also carried out cross-decoding analyses. We found that most of the regions exhibiting significant pattern difference decoding also showed a drop in cross-decoding for category across variation in task. However, a similar cross-decoding drop was not found in cross-decoding for task across variation in category. This is likely due to the difference between the cross-decoding and the pattern difference decoding approaches. While pattern difference decoding is related to testing for a drop in cross-decoding performance (for instance, a highly interactive neural code would be expected to generalize poorly), they are not identical and can dissociate. For example, a region could show significant pattern difference decoding without a cross-decoding drop in the presence of interaction effects small enough not to affect the classifier’s performance (seeFig. 2for an illustration). This may explain why we see cross-decoding drop of category across tasks but not cross-decoding drop of task across categories. Meanwhile, when positive results are obtained with the cross-decoding approach, it is highly correlated and consistent with those found in the pattern difference analysis. It is worth noting that despite the drops observed, cross-decoding performance remained above chance in the same regions, suggesting the coexistence of both generalizable and interactive components of the neural code, in the same way that a main effect and an interaction effect can coexist.

Although nonlinear mixed selectivity tuning has so far only been reported in PFC and PPC, interestingly, in the present study, we also found significant nonlinear mixed decoding in ventral areas. Given that task was decodable in ventral regions in the present study, it is possible that such decoding is evoked to support the representation of tasks and their interactions with object categories. Additional work is needed to establish whether such coding occurs due to intrinsic computations within the ventral pathway, or due to feedback from frontoparietal regions to ventral cortex (e.g.,Siegel et al., 2015;Xu, 2023).

Since nonlinear mixed selectivity can reflect either a rotation or a distortion of representational geometry across different contexts, we used RSA to characterize how the nonlinear mixed selectivity we observed is manifested in each region’s representational geometry. Using MDS, we first examined object category representational geometry in individual ROIs. We found that in early visual and ventral areas, object category played the dominant role in shaping representational geometry, but in dorsal areas both category and task jointly shaped the representation geometry. This echoes the results first reported byVaziri-Pashkam and Xu (2017)and shows that task is still prominently represented in dorsal areas even when the overall attended visual features are equated between tasks. Critically, we found that in IPS2-4, but not in V1–V4 or LOT/VOT, category representational geometry changed significantly between tasks, with IPS2-4 further showing a significant correlation between the magnitude of this change and the magnitude of pattern difference decoding. Thus, the nonlinear mixed selectivity coding for category and task we observe in IPS2-4 appears to be driven not by a mere rotation or shift of the representational geometry, but by an actual modulation of the structure/content of the geometry. By contrast, nonlinear mixed selectivity coding in LOT/VOT is consistent with a between-task rotation or shift in representational geometry without a change in the structure/content of the geometry. These results thus bring novel insights regarding how task may differentially impact object representations in ventral and dorsal regions.

In sum, this study replicates past work demonstrating that robust category information exists in both the ventral and dorsal pathways, and that dorsal pathway regions exhibit especially strong task modulation relative to ventral and early visual regions, even when tasks are matched in their spatial, object-based, and feature-based attentional demands. More importantly, we demonstrate the possibility of noninvasively measuring nonlinear mixed selectivity tuning for object and task information in the human brain for the first time, finding it in both ventral and dorsal visual regions, and linking it to a change in the representational geometry in dorsal but not ventral regions. The approach we use can be applied not just to combinations of task and stimulus variables as we have done here, but in principle to any pair of experimental variables employed in a factorial design, opening the door to broadly testing for this neural coding motif across the human brain.

## Data Availability

Extracted beta values, stimuli, and analysis code are freely available via the Open Science Framework athttps://osf.io/zvfxu/.
